# Salivary Biomarkers as Predictors of Obesity and Intermediate Hyperglycemia in Adolescents

**DOI:** 10.3389/fpubh.2022.800373

**Published:** 2022-06-10

**Authors:** Hend Alqaderi, Fahad Hegazi, Fahd Al-Mulla, Chung-Jung Chiu, Alpdogan Kantarci, Ebaa Al-Ozairi, Mohamed Abu-Farha, Saadoun Bin-Hasan, Aishah Alsumait, Jehad Abubaker, Sriraman Devarajan, J. Max Goodson, Hatice Hasturk, Mary Tavares

**Affiliations:** ^1^Department of Biochemistry and Molecular Biology, Dasman Diabetes Institute, Kuwait City, Kuwait; ^2^Kuwait School Oral Health Program, Ministry of Health, Kuwait City, Kuwait; ^3^Department of Oral Health Policy and Epidemiology, Harvard School of Dental Medicine, Boston, MA, United States; ^4^Department of Preventive Dental Science, Imam Abdulrahman Bin Faisal University, Dammam, Saudi Arabia; ^5^Center for Clinical and Translational Research, The Forsyth Institute, Cambridge, MA, United States; ^6^Department of Medicine, Faculty of Medicine, Kuwait University, Kuwait City, Kuwait; ^7^Farwaniya Hospital, Ministry of Health, Sabah Al Nasser, Kuwait; ^8^Department of Health Policy and Health Services Research, Boston University Henry M. Goldman School of Dental Medicine, Boston, MA, United States

**Keywords:** inflammation, saliva, metabolic disease, cytokines, children, obesity, insulin, C-Reactive Protein

## Abstract

**Introduction:**

Childhood obesity presents a major risk for metabolic diseases in adulthood. Noninvasive methods are needed for predicting the course of obesity in children and its complications. Using blood for longitudinal analyses of biomarkers to predict disease in children is not a convenient method. Saliva presents a noninvasive platform to detect inflammatory changes in biomarkers as possible predictive measures of future pathological events.

**Objectives:**

The aim of this study was to evaluate the relationship between specific salivary biomarkers, obesity, and intermediate hyperglycemia in children. We also investigated the longitudinal association between the salivary biomarkers and change in Body Mass Index-for-age percentile scores (BMIz).

**Methods:**

Data on 353 adolescents were collected from the individuals recruited for seven years in an ongoing Kuwait Healthy Life Study cohort. BMIz was measured at 10, 12, and 17 years of age. Interleukin (IL)-6, IL-8, IL-10, Leptin, C-Reactive Protein (CRP), Insulin, Vascular Endothelial Growth Factor (VEGF), and Monocyte Chemoattractant Protein-1 (MCP-1) were measured in saliva and serum. Additionally, fasting blood plasma glucose levels were recorded. Multilevel longitudinal regression modeling, mediation analyses, and logistic regression were used to determine the predictive value of salivary biomarkers in obesity and hyperglycemia.

**Results:**

Longitudinal analyses showed that with each one-unit increase of salivary CRP and insulin, there was a 3.5 kg/m^2^ and 3.2 kg/m^2^ increase in BMIz, respectively. Comparable to serum CRP and insulin, higher salivary CRP and insulin OR 4.94 [95%CI: 1.66,14., OR 2.64 [95%CI: 1.09, 6.38], respectively) were predictive of hyperglycemia and obesity (OR 4.53 [95%CI: 2.40,8.50], OR 3.29 [95%CI: 1.82,5.97], respectively). Insulin was a strong mediator in the relationship between obesity and hyperglycemia.

**Conclusion:**

Our findings demonstrated that salivary CRP and insulin were associated with hyperglycemia, obesity, and possibly diabetes in adolescents. Salivary biomarkers are a noninvasive approach with significant value for disease risk assessment and prevention.

## Introduction

Obesity is a strong risk factor for developing metabolic syndrome, diabetes, and cardiovascular diseases, with a resulting significant burden on the economy and health care system (1). Obesity is also a risk factor for major oral diseases, such as dental caries and periodontal disease (2, 3). Inflammation plays a critical role by triggering the metabolic signals in adipocytes in obesity (4). Emerging evidence indicates that increased circulating inflammatory cytokines lead to endothelial dysfunction and accelerate the development of diabetes and cardiovascular diseases (1, 5), which in turn could also lead to periodontal disease (3). Among the long list of potential “biomarkers” of obesity-associated inflammation, interleukin (IL)-6 (4, 6), IL-8 (7), IL-10 (6), leptin (4, 6), C-Reactive Protein (CRP) (4, 7), insulin (4), vascular endothelial growth factor (VEGF) (8), and monocyte chemoattractant protein-1 (MCP1) (7) were shown to be elevated in obese individuals while other markers such as adiponectin were reduced (6). In individuals without diabetes, inflammation reflected by increased CRP levels and insulin levels precedes glucose intolerance (9–11). Potentially, this would mean that early diagnosis of inflammation could be used to predict diabetes. There are no longitudinal studies on saliva and serum biomarker concentrations in adolescents.

Saliva testing has the advantage of being a quick, simple diagnostic tool compared to serum. Saliva presents a noninvasive platform to detect changes in biomarkers as possible predictive measures of future pathological events. Salivary biomarkers to predict and detect diabetes have not yet been explored and our study fills this gap in knowledge. To address this gap, we hypothesized that (1) certain salivary biomarkers for inflammatory conditions correlate with those in blood, (2) salivary biomarkers increase in individuals with obesity and intermediate hyperglycemia, and (3) salivary biomarker levels change with changes in BMIz (BMI-for-age percentile). Therefore, we aimed to evaluate the relationship of specific salivary biomarkers with obesity and intermediate hyperglycemia in a cohort of adolescents. We also investigated the longitudinal association between the salivary biomarkers and obesity.

## Materials and Methods

### Study Approval

This study was approved by the Dasman Diabetes Institute Human Ethical Review Committee in Kuwait and the Ministry of Health in Kuwait. We obtained informed consent from the parents or legal guardians and assent from the adolescents participating in the study at the outset. This study is reported in accordance with the guidelines of Strengthening the Reporting of Observational Studies in Epidemiology (STROBE) for observational clinical studies.

### Study Population and Sample Collection

This study analyzed the data collected from an earlier two-phase longitudinal study conducted by the authors of this report (12). In 2012, 8,317 primary school children, ages 9–11, were examined for both metabolic syndrome and oral disease (previously reported) (13) and surveyed; saliva samples were obtained (Visit 1). In 2014, 6,317 children from the same cohort (ages 11–13) were re-examined, surveyed, and saliva samples were obtained (Visit 2). In 2019, we were able to identify a subgroup (*N* = 353) of the Visit 2 cohort from two of the six governorates of Kuwait. They were enrolled and provided a third data time point (Visit 3). The students enrolled in these schools represent a wide variety of social class and ethnic groups among the Kuwaiti population. The selection into the cohort was not randomized across the population.

In Visits 1 and 2, only saliva samples were collected, while in Visit 3, both saliva and blood samples were collected. Therefore, correlations between salivary and blood biomarkers were evaluated for all the 353 participants in Visit 3. Matching saliva data from all three visits were available for 41 participants. They were used for longitudinal analyses.

The blood and saliva samples in all visits were obtained at the schools in the early morning after an overnight fast (≥12 h) and before breakfast. Unstimulated saliva samples were collected as previously described (13) and transferred to the lab on ice within an hour. The samples were processed at the Biobank Core facility laboratory and Biochemistry and Molecular Biology Department of the Dasman Diabetes Institute in Kuwait. The saliva samples were centrifuged at 2,800 rpm for 20 min at 4°C, after which the supernatants were transferred to a screw-cap 2D barcoded storage tubes (Thermo Scientific), and frozen at −80°C. Serum samples were collected using standard venipucture techniques in 7.5 mL red and black marbled topped tubes with clot activator (SST tubes) from 333 subjects. Blood was centrifuged at 3,000 RPM for 15 min at room temperature and serum samples were stored at −80°C.

### Saliva and Serum Analyses

The frozen samples were transported on temperature-monitored dry ice to The Forsyth Institute in Cambridge, MA, USA for biomarker analysis. On arrival, samples were thawed at 4°C overnight and kept on ice throughout the assay procedure. Insulin, CRP, adiponectin, leptin, IL-6, IL-8, IL-10, MCP-1, and VEGF were measured in 25 μl of saliva and serum samples using multiplex magnetic bead panels on a Luminex 200™ system (Luminex, Austin, TX) at the Forsyth Institute Multiplex Core Facility (Cambridge, MA). Results were evaluated using Bio-Plex Manager™ (Version 5.0; Bio-Rad, Hercules, CA).

### Glycated Hemoglobin A1c (HbA1c)

Glycated hemoglobin A1c (HbA1c) was analyzed at the Dasman Diabetes Institute. HbA1c was evaluated in the analysis as both a continuous and binary outcome. To create the binary variable, we used the American Diabetes Association cut-off values to represent the intermediate hyperglycemic state (14) that comprises borderline glycemia, as measured by an HbA1c > 5.6 % – <6.4% (15). Individuals with a confirmed diagnosis of diabetes (*n* = 6) were excluded from the analysis.

### Obesity-Associated Markers

Obesity was a dependent binary variable representing obese vs. non-obese adolescents using WHO criteria (16) in our analyses. Height was measured with a stadiometer, weight was measured with a calibrated digital scale, and Body Mass Index (BMI)-for-sex/age *z* score (BMI*z*) was calculated by computer using WHO guidelines (16). BMIz scores were categorized as obese, overweight, normal weight, and underweight (WHO 2020).

### Statistical Analysis

Salivary and serum biomarkers were analyzed as continuous and binary variables. The cut-off for the salivary binary variables was the 75th percentile of the continuous variable. The cut-off for serum binary variables was the 50th percentile of the continuous variable. We described continuous variables as means and standard deviations, and categorical variables as proportions and counts, stratified by intermediate hyperglycemic status or obesity. Due to right-skewed distributions of the biomarker levels, they were log10 transformed to approximally normal distributions before further analysis.

We conducted three modeling analyses in our study:

1. Multiple Binary Logistic Regression Modeling: We performed logistic analyses to test our hypothesis that levels of CRP, insulin, and adiponectin were associated with the probability of intermediate hyperglycemia or obesity.
2. Longitudinal Analysis: We performed repeated measurements multilevel analysis to evaluate if the BMIz changes were associated with the changes in the biomarker levels over time (three visits). Level 1 represented variations between children, and level 2 represented between-school variations. Since our primary outcome measure, BMIz, was a continuous variable with three values for each participant, we conducted multiple linear mixed models with random intercept and slope to evaluate the longitudinal change in the BMIz over the seven-year period (visit 1 in 2012, visit 2, in 2014, visit 3 in 2019). We used salivary levels of CRP, insulin, and adiponectin from the three visits as fixed effect variables, adjusting for baseline age and sex. Two indicator variables with Visit 1 as the reference were used for the “time” variable to represent the three visits. The school variable was used as a random intercept effect to account for within-school clustering. We also created an interaction variable between the main effect of each biomarker and the “time” variable.
3. Mediation Analysis: We conducted mediation analysis using the principles of Baron and Kenny (17) and the Structural Equation Modeling (SEM) (18) to evaluate if serum insulin is a mediator in the association between BMIz and intermediate hyperglycemia (details of mediation analyses in the supplementary).

We set the significance level at 0.05 and used STATA12 software (Stata Corp) in our data analyses.

## Results

Table 1 depicts the demographics of the individuals at three time points. At Visit 1, the mean age was 10 years, 12 years at Visit 2, and 17 years at Visit 3. The prevalence of obesity by BMIz scores increased from 26.5% at Visit 1 to 34.3% at Visit 3 over the 7 years.

**Table 1:** Demographics of individuals at three timepoints (SD, Standard Deviation).

	**Visit 1**	**Visit 2**	**Visit 3**
Total N	8,317	6,316	353
Age (in years) mean (SD)	10.0 (0.6)	12.0 (0.6)	17.0 (0.5)
Male (n, %)	5,098 (61.3)	3,966 (62.8)	173 (49.0)
Female (n, %)	3,219 (38.7)	2,350 (37.2)	180 (51.0)
High blood pressure (n, %)	1,990 (23.9)	2,571 (40.7)	50 (14.3)
Underweight (n, %)	193 (2.3)	133 (2.1)	7 (2.0)
Normal weight (n, %)	4,129 (49.7)	2,477 (39.2)	153 (43.3)
Overweight (n, %)	1,791 (21.5)	1,495 (23.7)	72 (20.4)
Obese (n, %)	2,204 (26.5)	2,211 (35.0)	121 (34.3)

*BMIz categories (underweight, normal weight, overweight, and obese) were defined using WHO guidelines*.

*Blood pressure was defined using the International Diabetes Federation (IDF) criteria*.

Table 2 further demonstrates our sample characteristics stratified by obesity using the WHO definition.

**Table 2:** Demographics of individuals during the three visits stratified by obese and non-obese using WHO guidelines.

	**Total**	**Obese**	**Non-obese**	***P*-value**
**Visit 1 N(%)**	8,317 (100)	2,204 (26·5)	6,113 (73·5)	·
Age (Mean)	10·0	10·1	9·9	<0·0001
Sex N (%)				
Male	5,098 (61·3)	1,197 (54·3)	3,901 (63·8)	<0·001
Female	3,219 (38·7)	1,007 (45·7)	2,212 (36·2)	
High blood pressure N (%)	1,990 (23·9)	964 (43·7)	1,026 (16·8)	<0·001
Underweight N (%)	193 (2·3)	··	··	··
Normal N (%)	4,129 (49·7)	··	··	··
Overweight N (%)	1,791 (21·5)	··	··	··
Obese N (%)	2,204 (26·5)	··	··	··
**Visit 2 N(%)**	6,316 (100)	2,211 (35·0)	4,105 (65·0)	
Age (Mean)	12·0	12·0	12·1	0·69
Sex N (%)				
Male	3,966 (62·8)	1,251 (56·6)	2,715 (66·1)	<0·001
Female	2,350 (37·2)	960 (43·4)	1,390 (33·9)	
High blood pressure N (%)	2,571 (40·7)	1,372 (62·1)	1,199 (29·2)	<0·001
Underweight N (%)	133 (2·1)	··	··	··
Normal N (%)	2,477 (39·2)	··	··	··
Overweight N (%)	1,495 (23·7)	··	··	··
Obese N (%)	2,211 (35·0)	··	··	··
**Visit 3 (N%)**	353 (100)	121 (34·3)	232 (65·7)	··
Age (Mean)	17·0	17·1	17·0	0·34
Sex N (%)				
Male	173 (49·0)	63 (36·4)	110 (63·6)	0·41
Female	180 (51·0)	58 (32·2)	122 (67·8)	
High blood pressure N (%)	50 (14·3)	33 (66·0)	17 (34·0)	<0·001
Intermediate hyperglycemia N (%)^a^	28 (8·4)	15 (53·6)	13 (46·4)	0·03
Underweight N (%)	7 (2·0)	··	··	··
Normal N (%)	153 (43·3)	··	··	··
Overweight N (%)	72 (20·4)	··	··	··
Obese N (%)	121 (34·3)	··	··	··

^a^ *Intermediate hyperglycemia is HbA1c >5·6 mmol/L.*

*··, N/A*.

Table 3 shows the mean salivary levels of the analytes at the three visits and serum levels at the third visit.

**Table 3:** Serum and salivary analytes at three timepoints.

	**Visit 1**	**Visit 2**	**Visit 3**
	**(saliva, *n =* 815)**	**(saliva, *n =* 42)**	**(saliva, *n =* 352; serum, *n =* 332)**
**CRP Mean (SD)**			
Saliva (pg/mL)	2.22 (0.71)	2.57 (0.52)	2.56 (0.56)
Serum (pg/mL)	··	··	5.99 (0.74)
**Insulin Mean (SD)**			
Saliva (pg/mL)	1.74 (0.62)	2.48 (0.41)	2.46 (0.36)
Serum (pg/mL)	··	··	2.89 (0.30)
**Adiponectin Mean (SD)**			
Saliva (pg/mL)	3.57 (0.41)	3.84 (0.42)	3.72 (0.39)
Serum (pg/mL)	··	··	6.90 (0.29)
**Leptin Mean (SD)**			
Saliva (pg/mL)	0.39 (1.23)	2.21 (0.15)	2.24 (0.25)
Serum (pg/mL)	··	··	4.14 (0.51)
**IL-6 Mean (SD)**			
Saliva (pg/mL)	0.88 (0.49)	0.94 (0.60)	0.70 (0.41)
Serum (pg/mL)	··	··	0.04 (0.38)
**IL-8 Mean (SD)**			
Saliva (pg/mL)	2.74 (0.47)	3.10 (0.42)	2.67 (0.34)
Serum (pg/mL)	··	··	0.76 (0.23)
**IL-10 Mean (SD)**			
Saliva (pg/mL)	0.65 (0.42)	0.20 (0.31)	0.38 (0.33)
Serum (pg/mL)	··	··	0.17 (0.40)
**MCP-1 Mean (SD)**			
Saliva (pg/mL)	2.42 (0.29)	2.56 (0.26)	2.48 (0.33)
Serum (pg/mL)	··	··	2.44 (0.23)
**VEGF Mean (SD)**			
Saliva (pg/mL)	2.47 (0.30)	3.07 (0.21)	2.78 (0.28)
Serum (pg/mL)	··	··	1.82 (0.33)

*The biomarkers data were reported in the log form and only those that were detectable were included*.

*CRP, C-Reactive Protein; SD, Standard Deviation; IL-6, Interleukin-6; IL-8, Interleukin-8; IL-10, Interleukin-10; MCP-1, Monocyte Chemoattractant Protein-1; VEGF, Vascular Endothelial Growth Factor. ··, N/A*.

**Figure 1:**
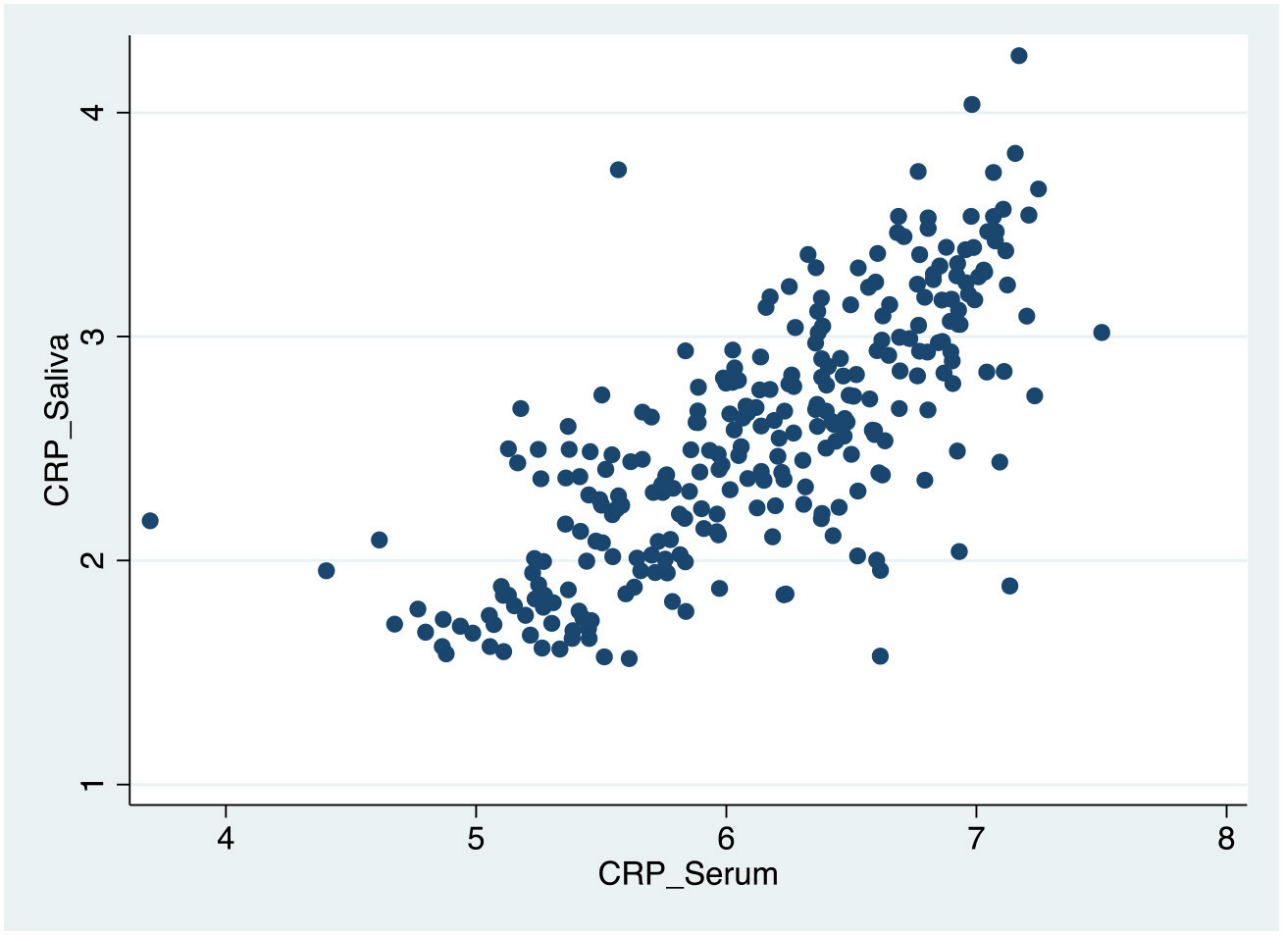
Scatter plot of the correlation between serum and salivary CRP biomarker levels.

**Figure 2:**
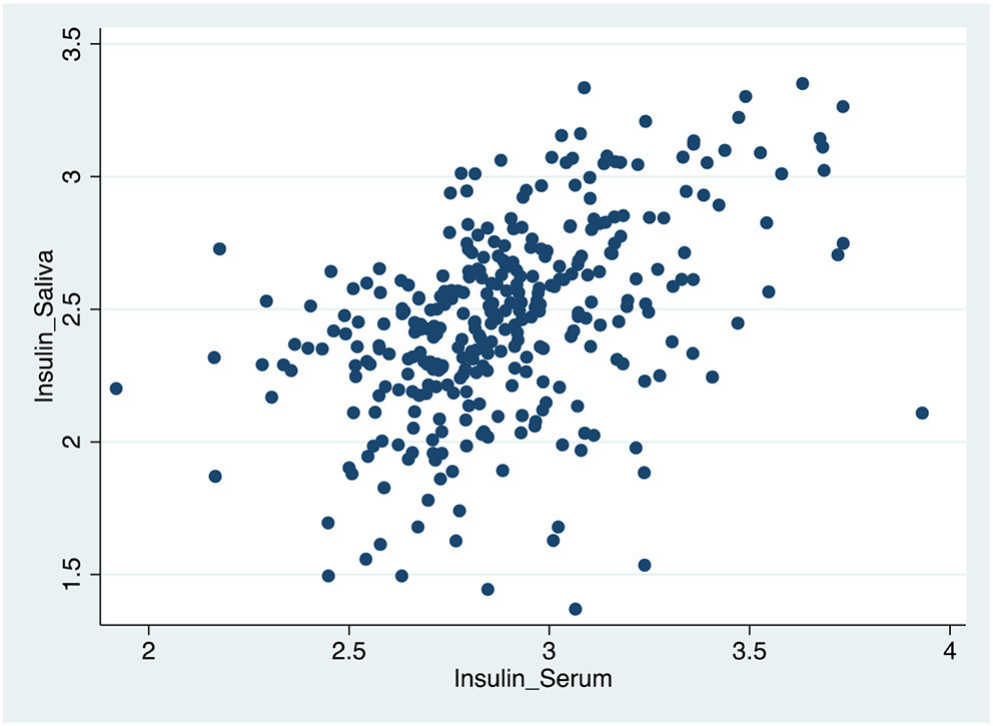
Scatter plot of the correlation between serum and salivary insulin biomarker levels.

**Figure 3:**
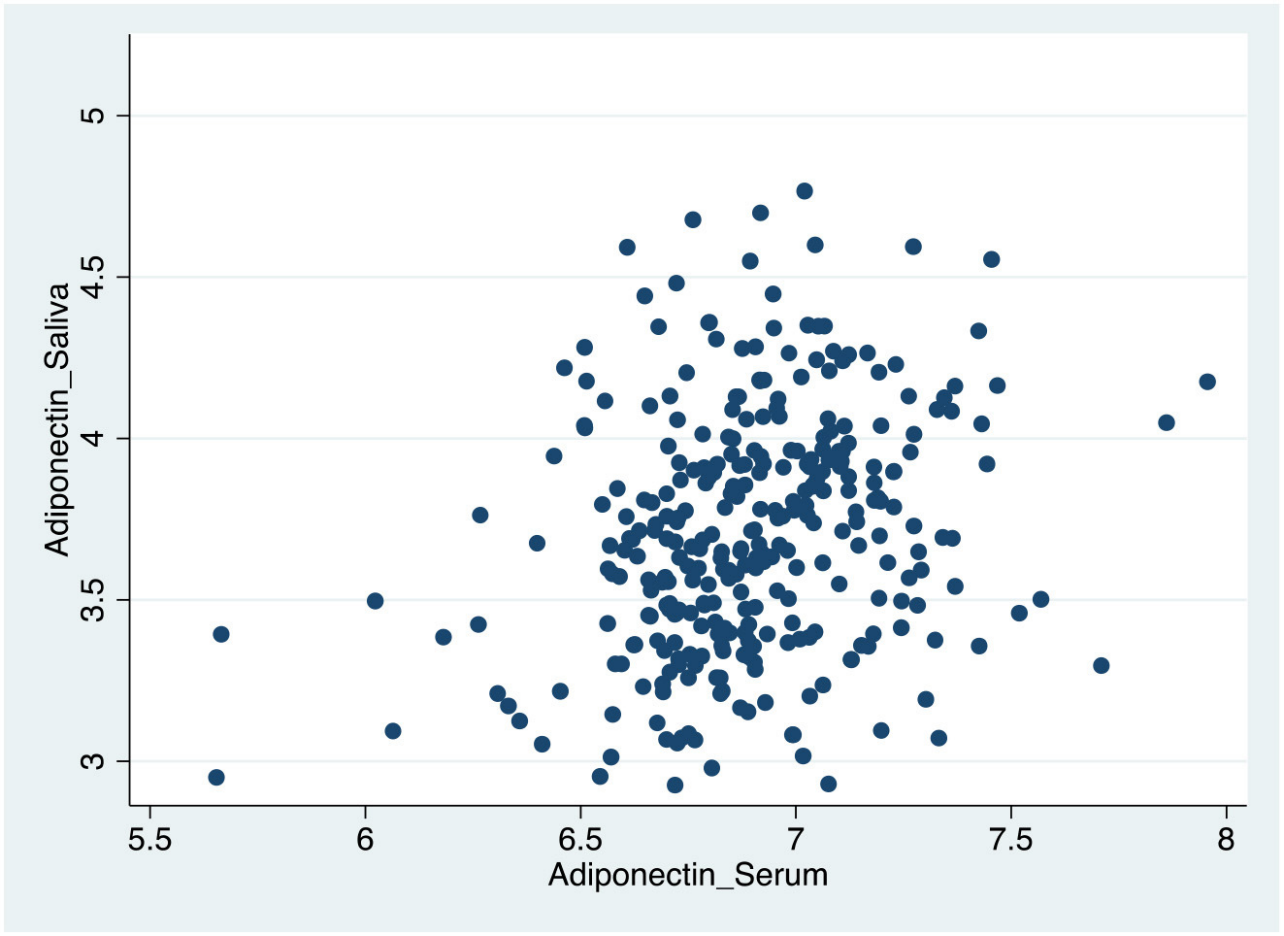
Scatter plot of the correlation between serum and salivary adiponectin biomarker levels.

Table 4, Figures 1–3 shows the Pearson correlation coefficients between the salivary and serum analytes at Visit 3. Insulin (r = 0.4, *P* < 0.0001), CRP (r = 0.7, *P* < 0.0001), and adiponectin (r = 0.2, *P* < 0.0001) were significantly correlated in saliva and serum. The detectable rates of the analytes are recorded in Table 3. CRP, insulin, and adiponectin had similar detectability rates (98.6, 98, 98.6%, respectively) in saliva and serum. While leptin had a lower detectability rate in saliva (80.7%) than serum (100%), others, such as IL-10, had a lower detectability rate in serum (7.2%) than saliva (62%).

**Table 4:** Pearson's correlation coefficients between serum and salivary biomarker levels at visit 3.

**Biomarker**	**Total N**	**Detectable**	**Total N**	**Detectable**	**Correlation**	***P*-value**
	**saliva**	**saliva N (%)**	**serum**	**serum N(%)**	**(R)**	
Insulin (pg/mL)	353	346 (98.0)	332	331 (99.7)	0.4	<0.0001
CRP (pg/mL)	353	348 (98.6)	332	332 (100)	0.7	<0.0001
Adiponectin (pg/mL)	353	348 (98.6)	332	332 (100)	0.2	<0.0001
Leptin (pg/mL)	353	285 (80.7)	332	332 (100)	0.08	0.1
IL-6 (pg/mL)	353	352 (99.7)	332	261 (78.6)	0.04	0.5
IL-8 (pg/mL)	353	316 (89.5)	332	331 (99.7)	0.003	0.9
IL-10 (pg/mL)	353	219 (62.0)	332	24 (7.2)	0.1	0.7
MCP1 (pg/mL)	353	348 (98.6)	332	332 (100)	0.08	0.1
VEGF (pg/mL)	353	341 (96.6)	332	332 (100)	0.05	0.3

*CRP, C-Reactive Protein; IL-6, Interleukin-6; IL-8, Interleukin-8; IL-10, Interleukin-10; MCP-1, Monocyte Chemoattractant Protein-1; VEGF, Vascular Endothelial Growth Factor*.

**Figure 4:**
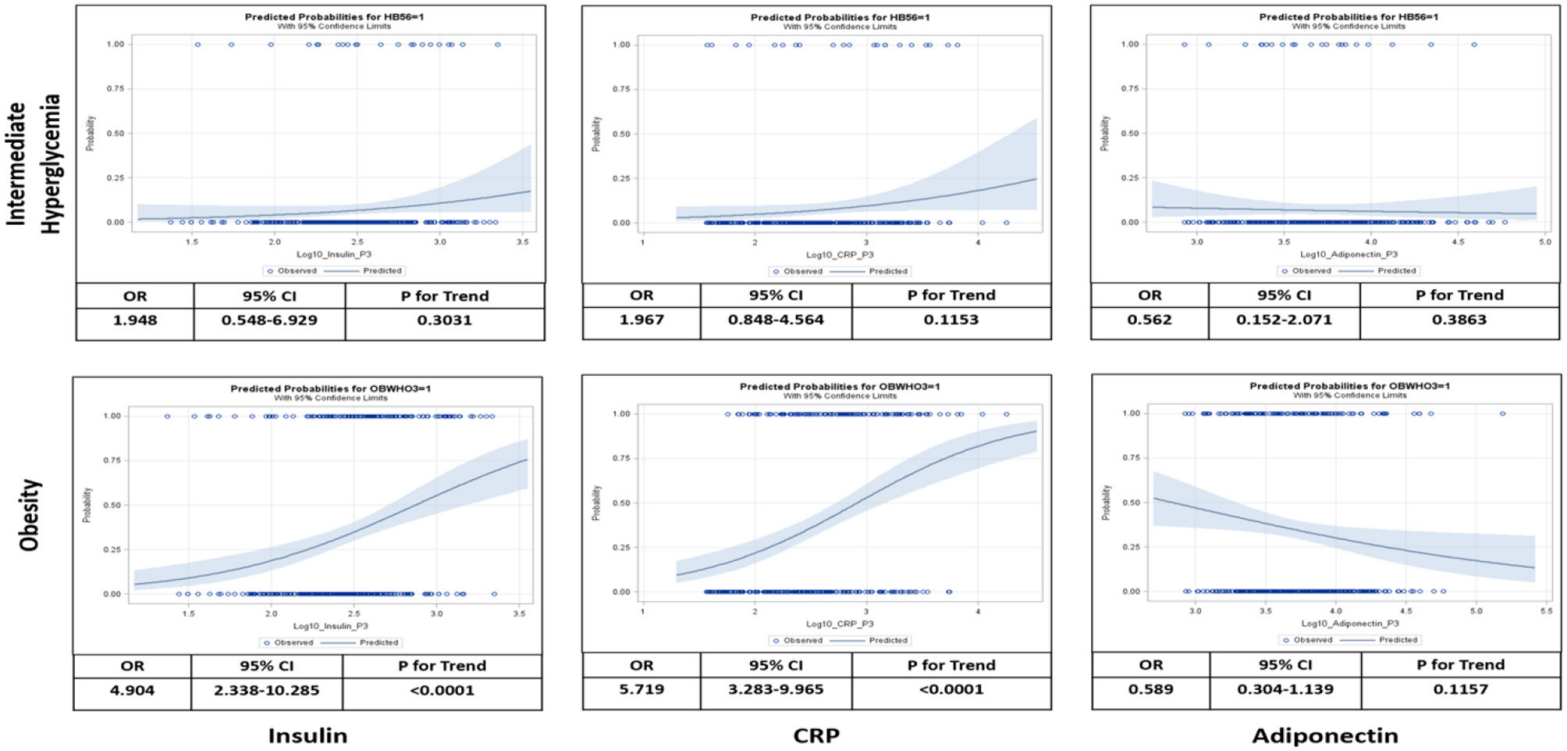
Logistic analysis relating salivary levels of insulin, C-reactive protein (CRP), and adiponectin to probabilities of intermediate hyperglycemia and obesity at visit 3. Intermediate hyperglycemia was defined as hemoglobin A1c (HbA1c) between >5.6– <6.4% and obesity was defined as body mass index (BMI) > [mean+2*standard deviation (SD)]. Odds ratio (OR), 95% confidence interval (CI), and test for linear trend were calculated as per 10-time increase of salivary biomarker levels adjusted for age, sex, blood pressure, and school.

Figure 4 shows a multivariate logistic regression of the three salivary biomarkers, CRP, insulin, and adiponectin, as continuous variables. The highest predicted probabilities of intermediate hyperglycemia and obesity occurred around the upper limits of CRP and insulin and the lower limits of adiponectin.

Table 5 show a multivariate logistic regression analysis between salivary and serum CRP, insulin, and adiponectin and their association with obesity and intermediate hyperglycemia after adjusting for age, sex, blood pressure, and school. Higher levels of salivary CRP and insulin (OR 4.97 [95%CI: 1.66,14.90]; *p* = 0.004, OR 2.64 [95%CI: 1.09,6.38]; *p* = 0.03, respectively) were associated with intermediate hyperglycemia. These findings were also similar for salivary CRP and insulin (OR 4.53 [95%CI: 2.40,8.50]; *p* < 0.001 OR 3.29 [95%CI: 1.82,5.97]; *p* < 0.001, respectively) in predicting obesity. Salivary adiponectin was only significant for predicting obesity (OR 0.54 [95%CI: 0.30,0.90]; *p* = 0.044). Similarly, serum CRP and insulin were associated with intermediate hyperglycemia (OR 3.35 [95%CI: 1.13,9.93]; *p* = 0.02, OR 5.93 [95%CI:1.82,19.34]; *p* = 0.003, respectively) and obesity (OR 14.75 [95%CI:7.35,29.60]; *p* = 0.001, OR 5.824 [95%CI: 3.24,10.45]; *p* = 0.001, respectively), and serum adiponectin was only associated with obesity (OR 0.43 [95%CI: 0.26,0.71]; *p* = 0.001) and was not associated with intermediate hyperglycemia (OR 0.76 [95%CI: 0.31,1.89]; *p* = 0.5). All the above models demonstrated acceptable degree of goodness of fit.

**Table 5:** Multiple logistic regression models for the associations of C-Reactive Protein, insulin, and adiponectin with intermediate hyperglycemia and obesity using data from visit 3.

	**Intermediate hyperglycemia**			**Obesity**		
	**OR**	***P*-value**	**95% CI**	**OR**	***P*-value**	**95%CI**
Salivary CRP	4·97	0·004*	1·66,14·90	4·53	<0·001*	2·4,8·5
Serum CRP	3·35	0·02*	1·13,9·93	14·75	<0·001*	7·35,29·60
Salivary insulin	2·64	0·03*	1·09,6·38	3·29	<0·001*	1·82,5·97
Serum insulin	5·93	0·003*	1·82,19·34	5·82	<0·001*	3·24,10·45
Salivary adiponectin	0·72	0·5	0·23,2·27	0·54	0·044*	0·3,0·9
Serum adiponectin	0·76	0·5	0·31,1·89	0·43	0·001*	0·26,0·71

*N = 323*.

*The models were adjusted for age, sex, blood pressure, and school (residential areas)*.

*Obesity was defined as BMIz> 2SD. Intermediate hyperglycemia was defined as hemoglobin A1c (HbA1c) between >5.6– <6.4%.*

*Salivary biomarker variables were stratified by 75^th^ percentile cutoff. Serum biomarkers variables were stratified by 50^th^ percentile cutoff (median).*

*OR, Odds Ratio; CI, 95% Confidence Interval; CRP, C-Reactive Protein.*

*, P-Value <0.05.

Mixed-effect linear regression modeling with BMIz as the outcome was utilized to investigate the longitudinal associations between BMIz and the salivary biomarkers, CRP, insulin, and adiponectin (Table 6). There was a statistically significant positive increase in the strength of the relationship between BMIz and salivary CRP level at Visit 3 compared to Visit 1 (β = 3.5, [95%CI: 1.9,5.07], *p* < 0.001), after adjusting for age and sex. Similarly, there was a statistically significant positive increase in the strength of the relationship between BMIz and salivary insulin level at Visit 3 compared to Visit 1 (β = 3.2, [95%CI: 1.30,5.10], *p* < 0.001). Although there was a statistically significant association between BMIz and salivary adiponectin across the three visits (β = −1.60, [95%CI: −2.80, −0.40], *p* = 0.007), we did not detect a longitudinal association (change over time) between BMIz and salivary adiponectin level.

**Table 6:** Multiple linear mixed models with random intercept and slope for the associations between BMI changes and the changes in the biomarker levels over time, adjusted for age and sex.

		**BMI**		
		**β coefficient (SE)**	***P*-value**	**95% CI**
Salivary CRP	Main effect	2.40 (0.48)	<0.001*	1.40 , 3.30
Salivary CRP ^a^interaction with time	CRP#time 2	−0.35 (1.03)	0.7	−2.30, 1.60
(salivary CRP at phase 1 is the reference)	CRP#time 3	3.50 (0.79)	<0.001*	1.90 , 5.07
Salivary Insulin	Main effect	2.10 (0.53)	<0.001*	1.10 , 3.10
Salivary insulin interaction with time	Insulin#time2	1.60 (0.95)	0.08	−0.19 , 3.50
(salivary insulin at phase 1 is the reference)	Insulin#time3	3.20 (0.96)	0.001*	1.30 , 5.10
Salivary Adiponectin	Main effect	−1.60 (0.6)	0.007*	−2.80, −0.40
Salivary Adiponectin interaction with time	Adiponectin#time2	−0.29 (1.15)	0.79	−2.50, 1.90
(salivary adiponectin at phase 1 is the reference)	Adiponectin#time3	−1.19 (1.27)	0.35	−3.60 , 1.30

*SE, Standard Error; CI, 95% Confidence Interval; CRP, C-Reactive Protein; #, the estimate for the beta coefficient of the interaction term variable is the multiple of the main effect with the variable ‘time'; the categorical variables are comprised of visit 1, visit 2, and visit 3 that show the rate of BMI change over time for salivary C-Reactive Protein, insulin, and adiponectin*.

^*^, P-Value <0.05. ^a^Interaction term variable is the multiple of the main effect with the variable ‘time'.

**Figure 5:**
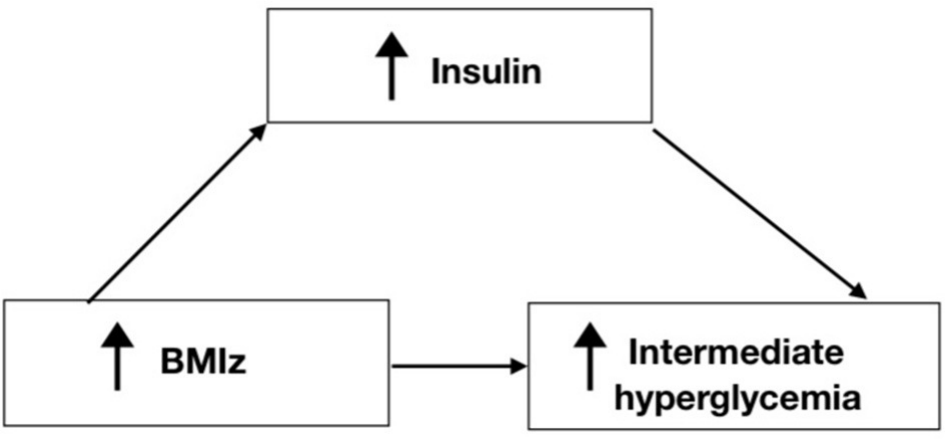
Pathway of the mediation process for Insulin in the relationship between BMIz and Intermediate hyperglycemia.

Table 7, Figure 5 shows the results of the mediation analysis which supports the full mediation effect of insulin in the relationship between increased BMIz and intermediate hyperglycemia.

**Table 7:** Mediation effect of insulin on the association between BMI and intermediate hyperglycemia adhering to the principles of Baron and Kenny and by using the standard equation modeling analysis, adjusted for age and sex.

**Baron and Kenny mediation analysis**									
	**Model 1**			**Model 2**			**Model 3**		
	**Intermediate hyperglycemia (HbA1c** **>** **5.6– < ** **6.4%)**			**Serum insulin**			**Intermediate hyperglycemia (HbA1c** **>** **5.6– < ** **6.4%)**		
	**OR**	***P*** **-value**	**95% CI**	**OR**	***P*** **-value**	**95% CI**	**OR**	***P*** **-value**	**95% CI**
Obese vs. non-obese	3.39	0.008*	1.30,8.30	5.40	<0.001*	3.20,9.09	2.20	0.09	0.80,5.80
Serum insulin	··	··	··	··	··	··	3.60	0.03*	1.10,11.70
**Standard equation modeling analysis**			
	**Estimate (bootstrap SE)**	**95% CI**	***P*** **-value**
Direct effect	−0.00045 (0.0006)	−0.0016, 0.00077	0.47
Indirect effect	0.0008 (0.00035)	0.000094, 0.0014	0.02*
Total effect	0.00033 (0.0004)	−0.00047, 0.0011	0.41
Ratio of the mediation effect (indirect to total effect)	0.0008/0.00033 = 2.4		
Ratio of indirect to direct effect	0.0008/−0.00045 = 1.7		
Ratio of total to direct effect	0.00033/-0.00045 = 0.73		

*Serum insulin were stratified by 50th percentile cutoff (median)*.

*The standard equation modeling calculated the confidence intervals and standard errors were from 5,000 random samplings with replacement by the bootstrapping method*.

*OR, Odds Ratio, CI, 95% Confidence Interval, SE, Standard Error*.

^*^P-value <0.05. ··, N/A.

## Discussion

This study provides important longitudinal data linking the development of hyperglycemia through CRP and insulin salivary inflammatory biomarkers. Out of the nine salivary cytokines we analyzed, CRP and insulin levels strongly differentiated obese vs. non-obese youths and individuals with intermediate hyperglycemia vs. those with normal glycemia. The same relationships were confirmed in serum but with different magnitudes. While significant correlations between saliva and serum exist in CRP, insulin, and adiponectin, the latter exhibits the weakest correlation. Salivary CRP and insulin were significantly associated with intermediate hyperglycemia at the 75th percentile cutoffs suggesting that a predictive value of the biomarkers such as levels found in serum could be indicative of diseases. Finally, longitudinal changes in BMIz show that with each one-unit increase of salivary CRP and salivary insulin, there was a 3.5 kg/m^2^ and 3.2 kg/m^2^ increase in BMIz over seven years. Our study sample were young people (mean age = 17 years old) free from diabetes and cardiovascular diseases. Hence, cytokine CRP and insulin expression in saliva at this age could potentially predict future progression to serious metabolic diseases, such as diabetes and cardiovascular diseases (5, 6).

CRP is a clinical marker for inflammation and is synthesized in the liver in response to IL-6, a pro-inflammatory cytokine (19). As a result, CRP increases during certain inflammatory conditions, such as rheumatoid arthritis, metabolic syndrome, and cardiovascular disease (19, 20). While it is known that inflammation plays a role in type 2 diabetes mellitus (21), recent data suggest that inflammation indexed by CRP precedes progression to type 2 diabetes (10, 11). This is shown in a study conducted among 4,101 healthy Japanese individuals without diabetes and found that a significant association was observed between elevated serum CRP levels and pre-diabetes, with the inflammation event preceding the progression to type 2 diabetes (10). Another study that compared individuals who were either healthy, prediabetics, or diabetics showed that inflammation appears to peak in the pre-diabetes group (11). These studies show that CRP acts as a predisposing factor in the progression of diabetes. Thus, it was suggested that inflammation should be targeted to prevent glucose intolerance and the consequent development of diabetes (9). The findings in our study support previous evidence that CRP affects blood glucose levels and BMIz that can increase the risk of diabetes. While CRP is usually assessed by serum tests, less invasive salivary testing for CRP has garnered some attention. Our study and others have found a direct positive correlation between CRP in serum and saliva (22–25). These positive correlations have been noted in neonates (23), children (25), as well as adolescents (22). Moreover, we showed a significant longitudinal increase in salivary CRP with increased BMIz over 7 years. This shows that saliva testing for CRP can potentially be used early in life as an alternative and less invasive measure for metabolic syndrome.

Hyperglycemia is associated with hyperinsulinemia, defined as increased insulin serum concentration and insulin resistance (26). There has been some debate regarding the role hyperinsulinemia plays in the incidence of type 2 diabetes; whether hyperinsulinemia causes insulin resistance leading to diabetes, or insulin resistance causes hyperinsulinemia. Some studies suggested that hyperinsulinemia leads to insulin resistance and type 2 diabetes and that it plays a greater role in inducing type 2 diabetes than hyperglycemia (26). One study has suggested that the increased insulin concentration is a significant independent predictor of type 2 diabetes even after accounting for adiposity and insulin resistance (27). However, other studies have shown that insulin resistance leads to hyperinsulinemia (28). Regardless of which causes the other, hyperinsulinemia sustains insulin resistance (26). The studies that investigated the role of hyperinsulinemia during obesity show that it could be the primary causative factor leading to both obesity and type 2 diabetes (29). Taken together, increased concentration of insulin precedes diabetes and could be a predictor for type 2 diabetes. Also, increased insulin could cause obesity rather than a product (29). Furthermore, the association between periodontal disease and obesity is mitigated by the development of insulin resistance (3). Our study results show that insulin was the sole mediator in the relationship between increased BMIz and intermediate hyperglycemia. Our mediation analyses showed that obesity had an indirect effect on intermediate hyperglycemia and that insulin mediates this relationship. Our findings support the fact that increased weight by itself cannot promote intermediate hyperglycemia and that it needs to be stimulated by insulin. In fact, by understanding that insulin mediates the process of the pathology of diabetes via obesity, the primary target of diabetes prevention in high-risk communities should be targeting insulin and other inflammatory biomarkers, allowing for early diagnosis and treatment.

Our study also showed a statistically significant correlation between insulin in saliva and insulin in serum. This correlation has also been observed by others (25, 30). While the concentration of insulin in saliva was shown to be 10 times lower than the concentration of insulin in serum, the correlation was statistically significant (30), which highlights the importance and value of saliva testing for insulin as a measure for early diagnosis and an outcome measure for therapeutic approaches.

The use of biomarkers in saliva to detect disease is still in the early stages, but the potential for its use is high. The gap between oral and systemic health still exists, but research in saliva testing to detect disease will help to bridge that gap. Finally, since individuals are encouraged to visit the dentist twice per year, dentists can collect and test saliva in patients who are at higher risk for developing both oral and systemic disease, manage the patients' oral disease and refer them to physicians to manage developing systemic diseases such as diabetes.

## Limitations

There are some limitations in this study that could be addressed with further research. Our study cohort was not a representative sample of the source population. Future studies should validate these findings in different populations with diverse exposure risks. Due to limited funding for Visit 3, we were able to enroll only 353 individuals from the 6,317 who participated in Visit 2.

Future studies in this area could focus on establishing a strategy based on preventing low-grade inflammation rather than just frequent screening for diabetes and late attempts to control hyperglycemia. To do this, research should aim to study salivary biomarkers as a less invasive method for diagnosing CRP and insulin levels to target high-risk individuals. More research is needed to determine predictive cut-off values for salivary biomarker levels to predict developing diseases such as intermediate hyperglycemia.

## Data Availability Statement

This study's data consist of de-identified participant data and are available from the corresponding author upon reasonable request. The data may also be obtained from a third party under the Dasman Diabetes Institute. The data are not publicly available. Requests to access the datasets should be directed to HA, hend_alqaderi@hsdm.harvard.edu.

## Ethics Statement

This study was approved by the Dasman Diabetes Institute Human Ethical Review Committee in Kuwait and the Ministry of Health in Kuwait. Written informed consent to participate in this study was provided by the participants' legal guardian/next of kin.

## Author Contributions

HA drafted the manuscript, contributed to the conception and design, data acquisition, and data analysis and interpretation. JG, MT, AK, and HH contributed to conception and design, data interpretation, and critically revised the manuscript. FH, EA-O, FA-M, SB-H, and JA contributed to the study design, data interpretation, and critically revised the manuscript. C-JC contributed to the data analysis and critically revised the manuscript. AA and SD contributed to the study design, data interpretation, and critically revised the manuscript. All authors gave final approval and agree to be accountable for all aspects of the work.

## Funding

This study was funded by the Dasman Diabetes Institute, Kuwait (Grant # RA/065/2011 and RA/005/2011; PI JG), Forsyth Institute, Cambridge, MA, USA (Grant # FSI-CP02; PIs MT and HH), and the Kuwait Foundation for the Advancement of Sciences, Kuwait (Grant # PR19-13MM-01; PI HA).

## Conflict of Interest

The authors declare that the research was conducted in the absence of any commercial or financial relationships that could be construed as a potential conflict of interest.

## Publisher's Note

All claims expressed in this article are solely those of the authors and do not necessarily represent those of their affiliated organizations, or those of the publisher, the editors and the reviewers. Any product that may be evaluated in this article, or claim that may be made by its manufacturer, is not guaranteed or endorsed by the publisher.
